# CAREGIVER OUTCOMES RELATED TO SLEEP DISTURBANCES IN PEOPLE LIVING WITH COGNITIVE IMPAIRMENT

**DOI:** 10.1093/geroni/igac059.2418

**Published:** 2022-12-20

**Authors:** Miranda McPhillips, Darina Petrovsky, Justine Sefcik, Junxin Li, Glenna Brewster, Nalaka Gooneratne, Subhash Aryal, Nancy Hodgson

**Affiliations:** University of Pennsylvania, Philadelphia, Pennsylvania, United States; Rutgers, The State University of New Jersey, New Brunswick, New Jersey, United States; Drexel University, Philadelphia, Pennsylvania, United States; Johns Hopkins University, Baltimore, Maryland, United States; Emory University, Atlanta, Georgia, United States; University of Pennsylvania, Philadelphia, Pennsylvania, United States; University of Pennsylvania, Philadelphia, Pennsylvania, United States; University of Pennsylvania, Philadelphia, Pennsylvania, United States

## Abstract

Sleep disturbances in people living with cognitive impairment (CI) may impose a great burden on caregivers. We examined the relationship between objective and subjective sleep measures in people living with CI and caregiver depression and mastery via a secondary analysis of The Healthy Patterns Clinical Trial (NCT03682185) baseline data (n=170). Objective sleep variables included total sleep time and sleep efficiency derived from 3 nights of actigraphy. Subjective sleep measures included PROMIS Sleep Related Impairment, Pittsburgh Sleep Quality Index, and Epworth Sleepiness Scale. Caregiver measures included Center for Epidemiological Studies Depression Scale and Caregiver Mastery Scale. People living with CI were female (67%), Black (80%), with mean age 73.4 ± 8.7. Caregivers were female (81%), family caregivers (80%) with mean age 56.5 ± 14.7. We used multiple regression analysis, adjusting for cognition, and examined if there were differences by caregiver gender. Poorer subjective sleep quality was significantly associated with more caregiver depression (B=0.407, SE= 0.198, p = 0.042). There were no significant sleep predictors for caregiver mastery; however, there was a moderating effect of gender on the association between subjective sleep quality and caregiver mastery. Female caregivers had increased caregiver mastery compared to males when the person living with CI had better sleep quality (B=0.555, SE= 0.218, p = 0.012). This study found that people living with CI sleep characteristics differentially influence caregiver outcomes. Sleep should be assessed using a combination of objective and subjective sleep measures in people living with CI to inform providers when interventions are needed.

